# Assessing and Improving Productivity in Primary Care: Proof of Concept Results for a Novel Value-Based Metric

**DOI:** 10.1007/s11606-024-08710-0

**Published:** 2024-06-26

**Authors:** Linda Diem Tran, Todd H. Wagner, Paul Shekelle, Karin M. Nelson, Stephan D. Fihn, Sydne Newberry, Ishita Ghai, Idamay Curtis, Lisa V. Rubenstein

**Affiliations:** 1 Health Economics Resource Center, Veterans Affairs Palo Alto Health Care System, Menlo Park, CA USA; 2 Stanford Surgery Policy Improvement and Education Center, Stanford Medicine, Stanford University, Stanford, CA USA; 3 VA Greater Los Angeles Healthcare System, Los Angeles, CA USA; 4 Department of Medicine, University of Washington, Seattle, WA USA; 5 Veterans Affairs Puget Sound Healthcare System, Seattle, WA USA; 6 Department of Health Systems and Population Health, University of Washington, Seattle, WA USA; 7 Geffen School of Medicine, University of California, Los Angeles (UCLA), Los Angeles, CA USA; 8 Fielding School of Public Health, UCLA, Los Angeles, CA USA; 9 RAND Corporation, Santa Monica, CA USA; 10 Pardee RAND Graduate School, RAND Corporation, Santa Monica, CA USA; 11 Division of General Internal Medicine & Health Services Research, Department of Medicine, University of California, Los Angeles (UCLA), Los Angeles, CA USA

## INTRODUCTION

Primary care is experiencing continuously increasing expectations regarding the quantity of care it should provide, and simultaneous pressure to deliver on competing demands, including rapid access to clinicians and high-quality clinical care. Interprofessional team-based primary care models,^1,2^ such as the medical home, have been introduced during the past two decades to address these challenges, including in the Veterans Health Administration (VA)^3^ with the Patient Aligned Care Teams initiative. As yet, there is no established metric for supporting primary care productivity improvement efforts by linking investment in interprofessional primary care teams to the multi-faceted care product expected from their work. Our objective was to develop and test a multi-dimensional measure of productivity that could be implemented using existing data, and to test its properties for distinguishing primary care clinics in ways that had face validity for supporting productivity improvement.

Productivity measurement has multiple purposes. We aimed to develop a measure that could support practice-level improvement initiatives. Evidence has shown that primary care improvement initiatives that are not guided by value-aligned objective measures often fall short.^4^ Yet, current productivity measurement tools (e.g., Relative Value Units and panel sizes) focus on the quantity of the care produced rather than its value to patients or their clinicians.

The number, types, and work processes of a clinic’s healthcare team members account for most of the ongoing costs of primary care^5^ and substantially determine the quality and quantity of care delivered. However, no current approach generates an integrated summary of productivity that links the investment in primary care teams to multiple value-based primary care products. What is needed is a productivity measure that integrates data on all (or as many as possible) of the Institute of Medicine domains of healthcare^6^ which can thus serve as a meaningful guide toward decision-making.

Our objectives in the work presented here were to (1) develop and test an econometrics-based measure for summarizing the efficiency with which primary care teams produced high-value care across their enrolled patient populations; (2) test the feasibility of calculating the measure on over 700 VA community-based outpatient clinics serving 3 million patients; and (3) assess proof of concept in terms of the measure’s feasibility, face validity, and potential for supporting primary care decision-making.

## METHODS

### Ethics

Our work used standard VA healthcare system administrative data that are continuously monitored for adherence to ethical and privacy regulations. Our project was conducted as quality improvement under the purview of VA’s national Office of Primary Care.

### Sample

The initial sample included 754 stand-alone VA community-based outpatient clinics. We excluded 19 (3%) clinics for which data were incomplete or unavailable due to small sample sizes, 16 (2%) clinics for which provider workload data were extreme or not plausible (< 250 patients served; < 150 patients or > 4000 patients per provider), and an additional 16 (2%) clinics located outside the contiguous US that operate in unique environments (e.g., availability of non-VA care or transportation). The remaining 703 (93%) clinics were included in analyses.

### Data Sources

All data used were from fiscal year (FY) 2019. For *clinical quality of care*, we used Healthcare Effectiveness Data and Information Set data reported on VA’s Electronic Quality Measurement platform.^7^ For *access and patient experiences of care*, we used VA’s Survey of Healthcare Experience of Patients (SHEP). SHEP surveys are based on Consumer Assessment of Healthcare Providers and Systems^8^ Surveys and are administered to randomly selected Veterans within each clinic after a primary care visit. For primary care *interprofessional team clinical time*, we used VA Primary Care Management Module (PCMM) tables from the VA Corporate Data Warehouse (CDW). PCMM tables identify staff assigned to each primary care team and report staff fulltime equivalent (FTE) hours for direct patient care by primary care team role. For *total number of patients served*, we accessed active patient-provider assignments in nationally maintained PCMM data files to assess provider panel sizes. For *patient age* and *risk scores*, we used administrative data from CDW.

### Measures

All measures contribute to a summary technical efficiency score for each included community-based outpatient clinic. Technical efficiency scores measure primary care clinics’ abilities to maximize outputs given their inputs. The score is calculated based on component measures—inputs and outputs—that comprise data aggregated across all of a clinic’s enrolled patients and/or all of its assigned providers.

#### Input Measures

To measure *interprofessional team clinical time*, we summed *FTEs* for all providers (physicians, nurse practitioners, and physician assistants), registered nurses or care managers, medical assistants or licensed practical nurses, clerks, and pharmacists assigned to a primary care team. We included the clinical time of team members whose assignments to a primary care team were active within the study year.

#### Output Measures

The composite measure for *clinical quality* applied a previously validated method^9^ to rank health systems and included 12 widely used measures addressing prevention of cancer, and detection or management of chronic medical diseases or mental health conditions (Supplement Appendix 1). Composite scores with a reliability of 0.7 and above were retained for analyses and transformed to a 0–100 scale with higher scores indicating better clinical quality. For *access*, we used the existing validated access composite score (0–100) from the SHEP patient survey. For *patient experiences of care*, we developed a composite measure (0–100; alpha: 0.8) based on the SHEP score domains of communication (four questions), comprehensiveness (three questions), and coordination (three questions) (Supplement Appendix 2). For *total number of patients served*, we calculated the average number of unique Veterans assigned to the clinic (across individual provider panels) over the fiscal year. In VA, provider panels represent active patients who have seen their providers within the past 2 fiscal years. We then rescaled the measure to have a maximum of 100.

#### Context Adjustment Measures

We adjusted for each clinic’s proportion of patients over age 70, and proportion of patients with a Nosos risk score > 1. Nosos is based on the Centers for Medicare and Medicaid Hierarchical Condition Categories risk adjustment model,^10^ and a score above 1 represents a VA patient with higher-than-average expected costs relative to the national average for VA patients.^11^

### Analysis

Based on an evidence review and expert panel, we determined that Data Envelopment Analysis (DEA) was the optimal method among available choices for developing our prototype measure. DEA, introduced by Charnes, Cooper, and Rhodes,^12^ is a computational method used to compare the performance of “decision-making units” or clinics on maximizing outputs given their quantity of inputs, or conversely, producing outputs with the minimum number of inputs. While DEA has been used to assess relative efficiency across primary care settings, such as Federally Qualified Health Centers,^13,14^ to our knowledge, this method has not been used to support primary care clinic–level improvement.

DEA requires users to set parameters. First, we used an output-oriented analysis to encourage users to focus on output improvement. Second, we controlled for patient risk (Nosos) and proportion of patients over age 70 as two non-discretionary measures of patient health by including them directly into the DEA calculation.^15^ Third, we assumed a variable returns to scale in which increasing inputs can lead to disproportionate increases in outputs.^16^ This decision allows for larger clinics to disproportionately serve more patients, for example. Fourth, we constrained the weighting of outputs in the DEA calculation to (1) ensure that all outputs contributed to the efficiency score and to (2) reduce the possibility that a clinic that performed very poorly on any one of the outputs (e.g., clinical quality of care) could be judged as efficient.

We used the “Benchmarking” and “Boot” packages in RStudio 2023.06.1 to implement the DEA model and calculate technical efficiency scores with 2000 bootstrap replicates. These efficiency scores measure the degree to which clinics, given the numbers of team FTEs dedicated to care delivery, maximized primary care outputs (see Supplemental Appendix 3 for efficiency score calculation information). To examine whether results had face validity and could show paths for clinic improvement, we summarized the distributions of efficiency scores, examined associations between efficiency scores and outputs, and reviewed the efficiency scores of clinics with similar input levels. These analyses were completed using Stata 18.0.

## RESULTS

Table 1 shows the ranges, means, and medians for each of the components of our DEA model, including outputs, inputs, contextual adjustments, and technical efficiency scores. The median scores for each output were as follows: clinical quality = 57 (range 0–100), access = 55.3 (range 27–85.4), patient experience = 69.4 (range 49.6–84.1), and number of patients served = 3249 (range 287–30,621). Table 1 also illustrates the wide variation across clinics in terms of staffing. The median FTEs for each team member category were as follows: primary care provider = 3.1 (range 0.2–26.3), nurse = 3 (range 0–28.2), medical assistant = 3 (range 0–28.6), administrative staff = 2.9 (range 0–23.1), and pharmacist = 0.5 (range 0–10.8). The median number (not shown) of Veterans served per primary care provider (PCP) FTE was 996 (range 304–2326) and was higher among large clinics with 16.4 or more team FTEs (1049 Veterans) compared to smaller clinics with fewer than 9 team FTEs (936 Veterans).

**Table 1:** Summary of Output Scores, Inputs, and Context, FY2019 (*n* = 703)

	**Range**	**Mean (SD)**	**Median**
Outputs			
Total patient score	0.9–100	14.1 (12.6)	10.6
Total patients served	287–30621	4331 (3869)	3249
Clinical quality score	0–100	57.3 (13.1)	57.0
Patient experience score (0–100)	49.6–84.1	68.9 (6)	69.3
Access score (0–100)	27–85.4	55.2 (10.1)	55.3
Inputs			
Primary care provider FTE	0.2–26.3	4.2 (3.5)	3.1
Registered nurse FTE	0–28.2	4 (3.4)	3.0
Medical assistant FTE	0–28.6	4 (3.3)	3.0
Administrative staff FTE	0–23.1	3.5 (2.9)	2.9
Pharmacist FTE	0–10.8	0.7 (0.9)	0.5
PC practice context adjustment—patient health status			
Percent patients aged 70 and over	5.4–80.5	42.9 (10.5)	44.0
Percent patients with Nosos scores > 1	9.3–49.5	21.2 (5.6)	20.6
Technical efficiency score	1–1.8	1.3 (0.1)	1.3

In terms of context, the proportion of patients aged 70 and over served by each clinic ranged between 5.4 and 80.5% (median 44%) and the percent of patients expected to have higher-than-average costs ranged between 9.3 and 49.5% (median 20.6%).

Primary care clinics were considered “efficient” when, relative to all other clinics in the sample, they produced maximum outputs (i.e., the sum of weighted scores for access, clinical quality, patient experience, and number of patients cared for) given the number of team hours (FTEs) used to deliver those output results. Of the 703 clinics in the analytic sample, 10 (1.4%) received an efficiency score of 1 and were classified as efficient. This indicates that few community-based outpatient clinics in our sample achieved “maximum” efficiency (represented by the minimum efficiency score of 1). The remaining clinics within the sample did not achieve efficiency and had scores above 1 up to a maximum of 1.8. The median efficiency score was 1.3 (95% CI 1.21–1.27), which suggests that half of clinics in the sample, given their staffing levels, could increase their outputs by at least 30%.

**Figure 1:**
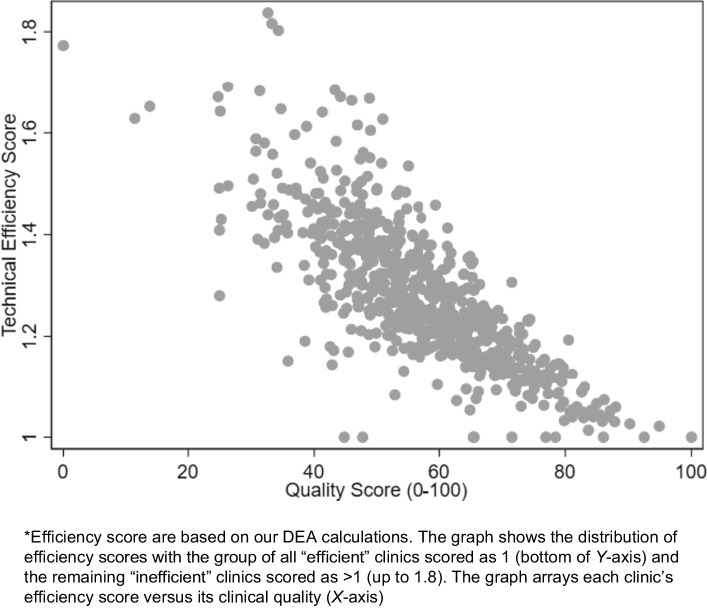
Scatterplot of the relationship between each primary care clinic’s efficiency score and clinical quality score, FY2019 (*n* = 703)*.

Figure 1 displays efficiency scores of 703 clinics in relationship to one primary care output (clinical quality). Efficiency scores are on the *Y*-axis and clinical quality scores are on the *X*-axis. The scatterplot shows that, as expected, scoring higher on clinical quality was strongly associated (*r* = − 0.8) with scoring lower (better) on efficiency score. We observed similar relationships between efficiency scores and the remaining outputs including access (*r* = − 0.5), patient experience (*r* = − 0.5), and total patients served (*r* = − 0.2). Serving greater proportions of older patients (*r* = 0.1) was correlated with greater inefficiency, although the relationship was weak.

**Figure 2:**
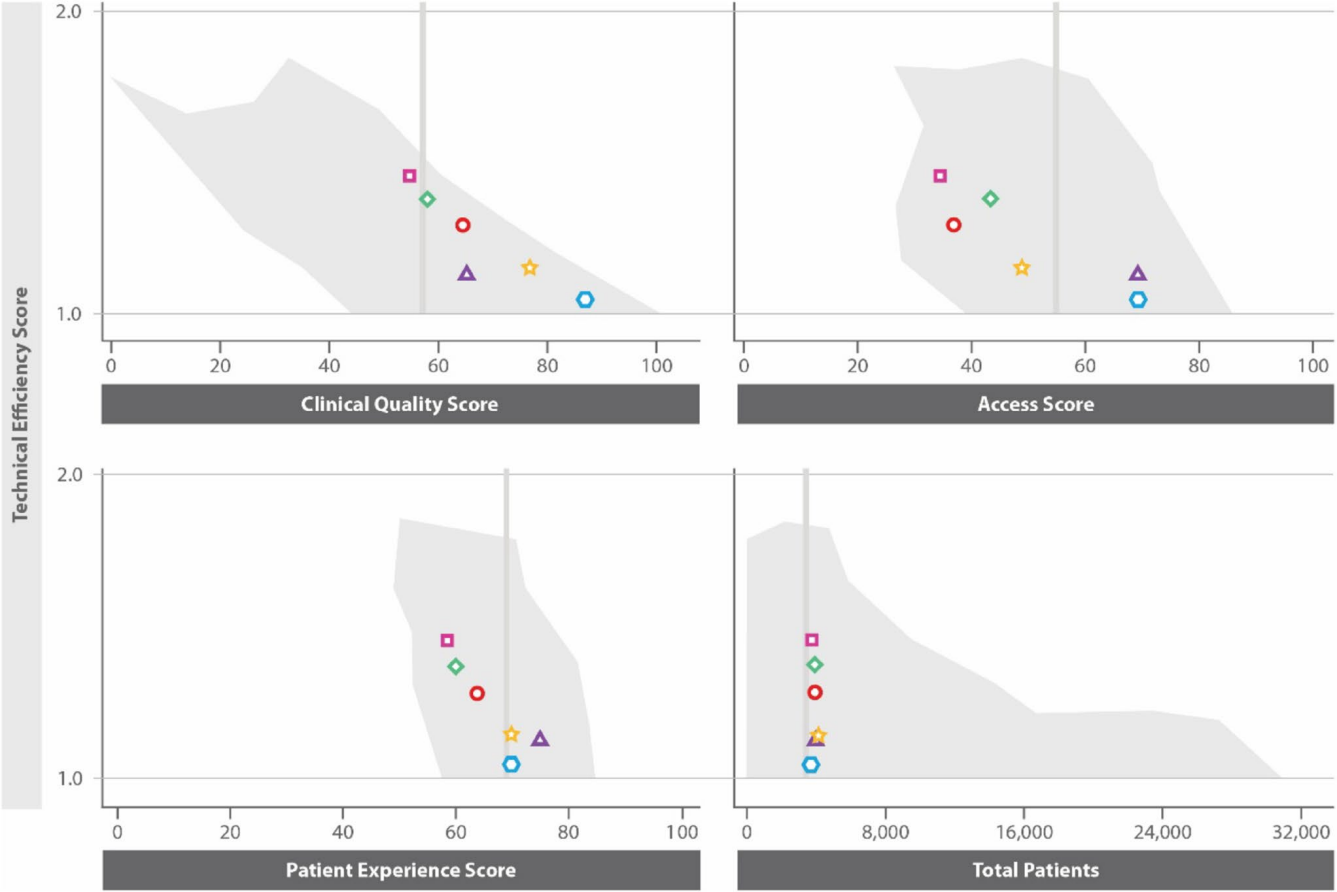
Display of primary care outputs (*X*-axis) versus technical efficiency scores (*Y*-axis)* for six example primary care clinics with similar staffing levels**, FY2019. *Efficiency scores are based on DEA calculations. Each of the four charts displays efficiency scores (*Y*-axis) versus scores for one of four outputs (*X*-axis). The grayed background shapes in each chart represent the distributions of efficiency scores versus each output score across all 703 primary care clinics. Vertical lines represent sample medians per output. **Each colored shape is assigned to a single clinic such that the clinic’s results can be visually tracked across each outputt. Efficiency scores are thus the same for each clinic across all four output charts, while output scores indicate where each “inefficient” clinic has room for improvement. Exact values for the six clinics are shown in Table 2.

In Fig. 2, we assessed clinic efficiency score results for all sample clinics relative to our four outputs. Each of the four charts in Fig. 2 displays the relationship between a clinic’s efficiency score (*Y*-axis) and its score for one of the outputs (*X*-axis). The distribution of values within each chart is depicted as a gray shadow. Figure 2 also highlights six example clinics that had similar staffing levels (Table 2). These clinics are marked across the four charts in differently colored symbols. Table 2 displays the input and output values for each of the six clinics. Although clinics had similar inputs (investment in staffing by team role), their performance across each healthcare domain varied significantly. Clinics that scored among the highest in outputs and performed better across multiple outputs were scored as more efficient. Based on total patients served alone, the Blue clinic would be rated below its peers. However, this clinic delivered exceptional clinical quality, while exceeding the sample median score for access, and achieved a nearly maximum overall efficiency score of 1.04. The Pink clinic scored below the median for both patient experience and access and was scored as less efficient. Green and Red clinics also scored low for access but scored higher in other domains and were scored as more efficient than the Pink clinic. The Orange and Purple clinics had comparable efficiency scores but different patterns of output scores.

**Table 2:** Efficiency Scores, Outputs, and Inputs for Selected Primary Care Clinics with Similar Inputs, FY2019*

			Selected clinic					
		Full sample medians	Blue	Orange	Purple	Green	Red	Pink
Efficiency score		1.3	1.04	1.14	1.14	1.38	1.29	1.45
Output scores	Clinical quality	57.0	87.0	77.7	65.4	58.7	64.4	54.7
	Access	55.3	69.6	48.5	69.2	41.3	38.7	37.2
	Patient experience	69.3	69.5	69.7	75.7	60.5	64.3	58.6
	Total patients	3249	3690	4066	3996	3959	3996	3935
Inputs (FTE)	PCP	3.1	4.0	4.0	4.0	4.0	4.3	4.0
	Registered Nurse	3.0	4.0	4.0	3.8	3.8	4.3	4.0
	Medical assistant	3.0	4.0	4.0	4.0	3.9	4.0	4.0
	Administrative staff	2.9	3.6	3.2	3.3	3.8	3.4	3.8
	Pharmacist	0.5	0.7	1.0	1.0	0.7	0.9	1.0

^*^Efficiency scores are based on DEA calculations. The six selected primary care clinics in this table are the same as those in Fig. 1. Maximum efficiency = 1; inefficiency increases as scores increase above 1

## DISCUSSION

As policymakers and healthcare delivery system leaders struggle to contain healthcare costs and deliver care efficiency, the lack of a measure for assessing primary care productivity relative to its value proposition is a potential threat to sustainment.^17–19^ We used an established method (DEA) to develop and test an innovative prototype measure that is driven by primary care values and goals. Our prototype, while still early in its development, holds promise.

First, the measure integrates four evidence-based, primary care outputs (clinical quality of care, access to care, patient experiences of care, and number of empaneled patients) into an efficiency calculation that accounts for the interprofessional teamwork hours that generated these outputs. Second, the measure proved feasible to implement using routine VA administrative data. Third, the measure identified substantial variation in efficiency across VA primary care outpatient clinics, a desirable characteristic for supporting improvement. As much as half of clinics had opportunities to substantively increase their outputs by at least 30%. Finally, the scores demonstrated face validity such that better performance on primary care outputs was associated with greater efficiency or productivity. Efficiency scores were strongly or moderately correlated with clinical quality, access, and patient experience scores. The correlation between efficiency scores and total patients served was low, which suggests that given staffing levels, clinics did not vary substantially in patients served to influence their efficiency scores. This is not surprising because VA issues guidelines on panel sizes. Importantly, clinics that performed better on outputs given the same level of team FTEs were scored as more productive than clinics with similar inputs but lower outputs. However, further development and testing will be required to establish full measure validity and utility.

Our approach is distinct from and more closely reflective of the primary care value proposition than are existing productivity-related measures.^20^ For example, other measures, such as Relative Value Units (RVUs), focus on the quantity but not the appropriateness of services provided, and can incentivize excessive visits, laboratory tests, or procedures while discouraging other critical but less easily documented primary care tasks.^21^ We observed that efficient or nearly efficient clinics did not have to score at the very top of all healthcare domains and that clinics can achieve comparable efficiency through varying output combinations. By incorporating four outputs, the prototype measure recognizes trade-offs in producing high-quality, accessible, and patient-centered care.

A strength of our analytical approach is its transparency. In future research, users can view all inputs and outputs that contribute to the efficiency score and provide feedback regarding accuracy. Additionally, alternative assumptions for conducting DEA calculations can be tested based on user input. For example, different weighting constraint choices can and should be tested to achieve the best match between efficiency scores and stakeholder values.

We were pragmatic in identifying a parsimonious group of available outputs that had well-demonstrated impacts on patient health outcomes. We did not attempt to test other domains of care such as continuity of care delivered. Thus, our prototype currently provides only a preliminary framework for supporting further development. Future work can examine other primary care outputs, to the degree that these outputs are meaningfully measurable in administrative data.

Our measure assumes all clinics could achieve all input-output combinations given appropriate management or decision-making.^16^ Yet, we saw that larger clinics may have been advantaged in terms of total number of patients served in relationship to inputs. Previous DEA research has addressed this issue by carrying out the analyses within sets of clinics grouped by key context factors such as size or rural or urban location;^13,14^ this solution is potentially feasible within VA, and future work is being designed to create meaningful peer groups.

Another limitation of our measure is that unnecessary referrals to specialty care or excessive emergency department care could be used by primary care teams to shift workload (and costs) to others while appearing to perform efficiently on our measure. A second efficiency model that focuses on total patient costs is one potential remedy for this issue. We see such model as secondary within our improvement use case, however, because many of the costs included will be from multi-factorial outcomes, such as hospitalization, that are not directly influenced by primary care team actions or decision-making.

Overall, our prototype measure aims to support primary care improvement and has both strengths and limitations for achieving this goal. A potential strength is that as a non-parametric method, DEA requires no assumptions about the distribution of efficiency across clinics or the relative importance of outputs and inputs, relying instead on observed results and user definitions to define maximum efficiency across the clinics in the sample. This gives DEA a strong basis in reality, but parametric methods that produce a ranked list of clinics may be more appealing to some stakeholders. Second, while our prototype met our goal of producing a summary efficiency score that can be broken down into its components, DEA output will require further tailoring to develop easily interpretable displays of results, and further testing of usability to determine whether and how clinics may use the score for decision-making. Third, because the model was developed within the framework of the VA primary care delivery system, and employed proprietary VA data, generalizability to other healthcare systems is uncertain. It should be noted, however, most of the data employed for both inputs and outputs are fairly standard and would likely be available elsewhere. Moreover, the data we used were substantially less complicated and potentially more available than, for example, those required by CMS to update and maintain the RVU system. Finally, and most importantly, we have not yet fully verified and validated our measure, nor evaluated its utility for clinic-level improvement.

## CONCLUSION

Our prototype efficiency measure shows promise for further development as a metric for supporting primary care improvement in relationship to a set of high-value outputs that are strongly linked to population health. We expect that measures meeting the requirements that have guided the development of our prototype can inform local primary care improvement, while also fueling a virtuous cycle of feedback to national healthcare system leaders about challenges and opportunities for increasing primary care efficiency relative to achieving multiple goals.

### Supplementary Information

Below is the link to the electronic supplementary material.

Supplementary file1 (DOCX 18 KB)

## Data Availability

Data used in this study have not been approved for public use.
