# Supplementary material for: Assessing and Improving Productivity in Primary Care: Proof of Concept Results for a Novel Value-Based Metric
Source: J Gen Intern Med. 2024 Jun 26;39(12):2317–23. doi: 10.1007/s11606-024-08710-0 (PMC11347497; doi:10.1007/s11606-024-08710-0)
Supplement: Supplementary file 1 — Supplementary file1 (DOCX 18 KB) [file 11606_2024_8710_MOESM1_ESM.docx]

**Appendix 1.** Outpatient Performance Metrics Selected for Clinical Quality Composite Development

| **Mnemonic** | **Description** |
| --- | --- |
| c9h_ec | Type 2 diabetes mellitus: outpatient – HbA1c annual |
| dmg23h_ec | Type 2 diabetes mellitus: hemoglobin A1c poor control |
| dmg27h_ec | Type 2 diabetes mellitus: blood pressure less than 140/90 |
| dmg34h_ec | Type 2 diabetes mellitus: medical attention for nephropathy – renal testing (outpatient) |
| ihd53h_ec | Controlling high blood pressure |
| ihd5h_ec | Hypertension: outpatient BP less than 140/90 age 18-59 |
| mdd43h_ec | Effective acute phase treatment (12 weeks) |
| mdd47h_ec | Effective continuation phase treatment (6 months) |
| statn1_ec | Statin therapy for patients with cardiovascular disease |
| statn4_ec | Statin adherence for patients with cardiovascular disease |
| statn7_ec | Statin therapy for patients with diabetes |
| statn8_ec | Statin adherence for patients with diabetes |

**Appendix 2.** Survey of Healthcare Experience of Patients (SHEP) Questions by Reporting Measure Domain

| **Domain** | **SHEP survey question** |
| --- | --- |
| **Access** | In the last 6 months, when you contacted this provider’s office to get an appointment for care you needed right away, how often did you get an appointment as soon as you needed? |
|  | In the last 6 months, when you made an appointment for a check-up or routine care with this provider, how often did you get an appointment as soon as you needed? |
|  | In the last 6 months, when you contacted this provider’s office during regular office hours, how often did you get an answer to your medical question that same day? |
| **Communication** | In the last 6 months, how often did this provider explain things in a way that was easy to understand? |
|  | In the last 6 months, how often did this provider listen carefully to you? |
|  | In the last 6 months, how often did this provider show respect for what you had to say? |
|  | In the last 6 months, how often did this provider spend enough time with you? |
| **Care coordination** | In the last 6 months, how often did this provider seem to know the important information about your medical history? |
|  | In the last 6 months, when this provider ordered a blood test, x-ray, or other tests for you, how often did someone from this provider’s office follow up to give you those results? |
|  | In the last 6 months, how often did you and someone from this provider’s office talk about all the prescription medicines you were taking? |
| **Comprehensiveness** | In the last 6 months, did anyone in this provider’s office ask you if there was a period of time when you felt sad, empty, or depressed? |
|  | In the last 6 months, did you and anyone in this provider’s office talk about things in your life that worry you or cause you stress? |
|  | In the last 6 months, did you and anyone in this provider’s office talk about a personal problem, family problem, alcohol use, drug use, or a mental or emotional illness? |

**Appendix 3.** Output-Oriented DEA Model

Efficiency scores are based on the weighted sum of inputs relative to the weighted sum of outputs. For each clinic, the solution determines weights for each input and output that, when applied to all clinics, minimizes the clinic’s score.

$$h_{k} = \min_{u, v} \frac{\sum_{i=1}^{m} v_{i}x_{i,k}}{\sum_{r=1}^{s} u_{r}y_{r,k}}$$

such that

$h_{k}\geq1$ for all clinics

$$v_{i}\geq0$$

$$u_{r}\geq0$$

Weight restrictions, if any, are met

where

$h_{k}$ is the relative efficiency of clinic *k*

$v_{i}$ is the weights to be determined for input *i*

*m* is the number of inputs

$u_{r}$is the weights to be determined for output *r*

*s* is the number of outputs
